# *Trichoderma asperellum* Inoculation as a Tool for Attenuating Drought Stress in Sugarcane

**DOI:** 10.3389/fpls.2021.645542

**Published:** 2021-04-15

**Authors:** Daniele Scudeletti, Carlos Alexandre Costa Crusciol, João William Bossolani, Luiz Gustavo Moretti, Letusa Momesso, Brenda Servaz Tubaña, Sérgio Gustavo Quassi de Castro, Elisa Fidêncio De Oliveira, Mariangela Hungria

**Affiliations:** ^1^Department of Crop Science, College of Agricultural Sciences, São Paulo State University, Botucatu, Brazil; ^2^School of Plant, Environmental, and Soil Sciences, LSU AgCenter, Baton Rouge, LA, United States; ^3^AgroQuatro-S Applied Agronomic Experimentation, Orlândia, Brazil; ^4^Embrapa Soybean, Londrina, Brazil

**Keywords:** Plant growth promoting fungus, low water availability, reactive oxygen species, antioxidant metabolism, Saccharum officinarum L.

## Abstract

Drought stress is an important concern worldwide which reduces crop yield and quality. To alleviate this problem, *Trichoderma asperellum* has been used as a plant growth-promoting fungus capable of inducing plant tolerance to biotic and abiotic stresses. Here, we examined the effect of *T. asperellum* inoculation on sugarcane plant above and belowground development under drought stress and investigated the role of this fungus on inducing tolerance to drought at physiological and biochemical levels. The experiment was performed in pots under greenhouse conditions, with four treatments and four replicates. The treatments consisted of sugarcane plants inoculated or not with *T. asperellum* and grown under drought stress and adequate water availability. Drought-stressed sugarcane plants inoculated with *T. asperellum* changed the crop nutrition and chlorophyll and carotenoid concentrations, resulting in increased photosynthesis rate, stomatal conductance, and water use efficiency compared to the non-inoculated plants. In addition, the antioxidant metabolism also changed, increasing the superoxide dismutase and peroxidase enzyme activities, as well as the proline concentration and sugar portioning. These cascade effects enhanced the root and stalk development, demonstrating that *T. asperellum* inoculation is an important tool in alleviating the negative effects of drought stress in sugarcane. Future studies should be performed to elucidate if *T. asperellum* should be reapplied to the sugarcane ratoons.

## Introduction

Drought stress is one of the major abiotic stresses worldwide affecting plant growth, development, and crop yield (Basu et al., 2016; Rosa et al., 2020). Recently, Gupta et al. (2020) reported that drought alone might cause more losses in crop yield than all plant diseases combined. Drought is defined as a stress condition in which there is a deficit of water in the soil, and consequently, there is an increase in soil temperature (especially during the day), a reduction in the nutrient availability, and, eventually, an increase in soil salinity (Tardieu, 2013; Tardieu et al., 2018).

Sugarcane (*Saccharum* sp.), the most important energy crop in the world, in general, is considered to be partial-drought sensitive (Marcos et al., 2018a). Grown under low water availability conditions, sugarcane often produces a significantly low stalk yield with poor technology quality, culminating in low sugar and ethanol production. Studies have shown that drought stress can induce several morphological, physiological, and metabolic responses of plants, resulting in reactive oxygen species (ROS) production, leading to increased cell damages and antioxidant enzyme inactivation (Vilela et al., 2017). In Brazil, with the expansion of sugarcane in the Midwest region, planting started to be done in a wide textural diversity and soil fertility (Marcos et al., 2018b). Soils with low fertility and, generally, sandy texture present themselves as limiting factors for the development of sugarcane (Bordonal et al., 2018). This occurs due to the low storage capacity of water and nutrients in the soil, especially in periods of high resource demand for the plant (Ribeiro et al., 2013; Marcos et al., 2018b).

As a semi-perennial crop, sugarcane experiences seasonal variations in water availability and higher temperatures (Marcos et al., 2018b), leading to loss of yield and quality of the final product, as well as a reduction in remuneration to the producer. Thus, drought stress seriously threatens the sugarcane production chain (Ribeiro et al., 2013), reducing the production of its derivatives and raising its prices in the domestic and foreign markets. Therefore, it is critically important to develop effective management practices to alleviate the negative impacts of drought stress on sugarcane growth and development (Fahad et al., 2017). Genetic breeding has sought to obtain new cultivars more resistant to drought (Mall et al., 2020); however, the establishment of management practices that increase the mitigation of environmental stresses is necessary.

An innovative technique increasingly studied and implemented in agriculture is the use of plant growth-promoting microorganisms (bacteria and fungi) to induce plant resistance to abiotic and biotic stresses (Zhang et al., 2016; Poveda, 2020). This approach is new and effective in increasing the plant tolerance to environmental climate challenges, which can play an important role in the development of sustainable agricultural systems (Rajesh et al., 2016). Among this group of microorganisms, *Trichoderma* spp. is a rhizospheric fungus of great agricultural and environmental importance, which can confer numerous beneficial effects in promoting plant growth and development (Harman et al., 2004), in inducing systemic resistance in plants (Kashyap et al., 2017; De Sousa et al., 2020) and as a biocontrol agent for fungal diseases (Harman et al., 2004; Mastouri et al., 2010). There are countless species within the genus *Trichoderma*, and a highlight has been given to *T. asperellum* (Harman, 2005; De Sousa et al., 2020; Zhao et al., 2020). Several authors have reported the ability of this group of microorganisms to relieve biotic and abiotic stresses of germinating plants and seeds, reducing the damage caused by ROS (Poveda, 2020). However, the underlying mechanisms responsible for ROS scavenging have yet to be elucidated (Kashyap et al., 2017). While most of the work is done on crops with a short growth cycle, little information is available on the potential of *T. asperellum* to act as plant growth promoters in increasing tolerance against drought stress, especially in a semi-perennial crop as sugarcane.

Based on these arguments, our study hypothesized that inoculation with *T. asperellum* may reduce the negative effects of drought on nutrition and physiological and morphological parameters of sugarcane plants. To test these hypotheses, our study evaluated the acquisition of nutrients, the concentration of photosynthetic pigments, gas exchange parameters, antioxidant metabolism, and root growth and stalk yield in sugarcane plants submitted to drought stress under application of the *T. asperellum* inoculant.

## Materials and Methods

### Growth Conditions

This study was conducted in an experimental greenhouse with a climate-controlled environment in Botucatu, São Paulo State, Brazil (22° 51′ S, 48° 26′ W, elevation 815 m above sea level). Greenhouse had an internal heating/cooling air circulatory system to maintain the temperature between 22 and 32°C and 70% of relative humidity. The experiment was performed using polyethylene pots with a capacity of 38 dm^3^ and 50 kg soil (soil density: 1.43 g cm^–3^). Each pot was filled with RedYellow Latosol soil previously air-dried and then mixed homogenously with lime (18 g 50 kg soil^–1^, aiming to obtain 70% of base saturation) according to the methodology proposed by van Raij et al. (1997). The pots with soil and lime were moistened and incubated for 30 days for the lime to react with the soil. Subsequently, the soil of each pot was homogenized individually with the fertilizers [8 g urea (45% N), 8.13 g potassium chloride (KCl; 60% K_2_O), 8.6 g triple superphosphate (42% P_2_O_5_ and 10% Ca), and 13.4 g micronutrients (2% Mn, 1% Cu, 10% Zn, and 0.2% Mo)], according to the soil physicochemical analysis (Kiehl, 1979; van Raij et al., 2001; Donagema et al., 2017; Supplementary Table 1). Fertilizer application occurred in order to mimic the management practices carried out in the cane fields by producers in Brazil. Sugarcane variety used was RB855536 (RIDESA BRASIL, Araras, Brazil), characterized as sensitive to water deficit (Hoffmann et al., 2008). The seedlings were produced by extracting a layer of reserve tissue and placing a bud per tube, filled with filter cake, substrate, and sand.

### Experimental Design and Treatment Establishment

**FIGURE 1:**
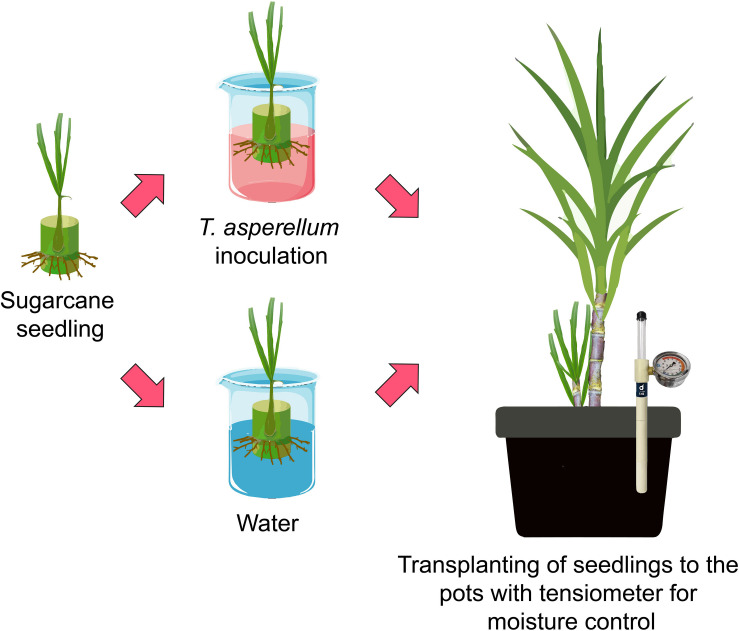
Schematic figure representing the experiment setup.

The experimental design was a completely randomized block with four replicates and a 2 × 2 factorial scheme. The treatments consisted of sugarcane seedlings inoculated with water (control treatment) or with *Trichoderma asperellum* (isolates-GF 332) and grown under conditions of adequate water availability (field capacity) and moderate drought stress [50% available water capacity (AWC)]. The biological inoculant was a commercial conidia-based formulation of *T. asperellum* strain GF 332 [media culture: synthetic polymer polyvinylpyrrolidone (PVP), 1 × 10^10^ CFU g^–1^ of granules, water dispersible granules (WG), Lallemand^®^, Patos de Minas, MG, Brazil). The inoculations were carried out according to the technical recommendations of the manufacturers. These products are registered with the Ministry of Agriculture, Livestock, and Food Supply of Brazil. After 20 days of the bud having been planted in the tubes, sugarcane seedlings were immersed in a solution containing an inoculant with *T. asperellum* (GF 332) to a concentration of 1.0 × 10^10^ CFU mL^–1^ and then transplanted to the experimental pots (Figure 1). From the soil used in this experiment, the soil water retention curve (SWRC) was obtained at 0.00–0.15-m depth (see details in Supplementary Material) and the amount of water to raise the soil to field capacity was calculated. The pots with sugarcane seedlings were kept in field capacity until the seedlings reached the phenological stage of tillering and canopy development (Bonnett, 2013). During this period, two top-dressing fertilizations were carried as follows: at 30 days after transplanting (DAT) [3.9 g urea (45% N), and 4.3 g KCl (60% K_2_O)] and at 115 DAT [15 g ammonium sulfate (21% N and 23% S), and 2.8 g KCl (60% K_2_O)]. At the beginning of the tillering and canopy development (∼120 DAT), treatments with drought stress started.

The management of irrigation occurred through the use of a vacuum tensiometer, which indicated the daily irrigation up to 0.15-cm depth, considering the SWRC (Supplementary Figure 1). The data obtained from the tensiometer were supplied in a digital spreadsheet that automatically calculated the volume of water to be applied to raise the soil’s potential to field capacity (−10 kPa as 100% AWC = control treatment) according to SWRC. Based on the SWRC, the amount of water needed to maintain moderate drought stress (50% AWC) was also determined. The tensiometer readings and irrigation occurred daily in the morning (8:00–10:00 h a.m.).

### Sugarcane Chemical and Physiological Parameters Assessed

All nutritional, biochemical, and physiological analyses occurred from sugarcane leaves [Leaf +1 or Top Visible Dewlap leaf (TDV)] collected during the grand growth phenological stage (Bonnett, 2013), at ∼60 days after beginning of the drought stress, collected between 9:00 and 10:00 h a.m.

#### Sugarcane Crop Nutrition

The samples from leaf +1 were dried and ground in a Willey-type mill with a 1-mm screen, and subsequently, the macronutrients were determined. All of the macronutrients (P, K, Ca, Mg, and S), except N, were extracted by nitroperchloric digestion and determined by atomic absorption spectrophotometry. Nitrogen was extracted through sulfuric digestion and determined by the Kjeldahl distillation method as described by AOAC (2016).

#### Photosynthetic Pigments

To determine the photosynthetic pigments (Chl *a*, Chl *b*, and carotenoids) in the leaf tissues, five discs were cut between the edge and the leaf central vein from the fresh leaf +1. The discs were kept for 24 h in glass vials with 2 mL of N,N-dimethylformamide (DMF), as proposed by Lichtenthaler (1987). The determination of Chl *a*, Chl *b*, and carotenoids occurred through readings in absorbance at wavelengths of 664, 647, and 480 nm, respectively. The occurred according to the methodology proposed by Wellburn (1994).

#### Gas Exchange

Sugarcane leaf gas exchange was assessed between 8:00 and 10:00 a.m. Leaf +1 was evaluated using a portable Infra Red Gas Analyzer CIRAS-3 Portable Photosynthesis System (PP Systems Inc., Amesbury, MA, United States) with photon irradiance of 1,200 μmol m^–2^ s^–1^. The variables assessed were net photosynthesis (*A*; μmol CO_2_ m^–2^ s^–1^), stomatal conductance (*gs*; mol H_2_O m^–2^ s^–1^), substomatal CO_2_ concentration (*Ci*; mmol CO_2_ mol^–1^ air), leaf transpiration (*E*; mmol H_2_O m^–2^ s^–1^), water use efficiency [WUE; μmol CO_2_ (mmol H_2_O) ^–1^], calculated by the *A*/*E* ratio, and carboxylation efficiency, calculated by the *A*/*C*i ratio.

#### Nitrate Reductase Activity

The *in vivo* nitrate reductase (NR) activity was adapted from Reis et al. (2009). Sugarcane fresh leaves (100 mg) were cut with scissors and transferred to assay tubes containing 3 mL of phosphate buffer solution pH 7.4 (50 mM L^–1^ plus 200 mM L^–1^ KNO_3_). These samples were vacuum-infiltrated for 5 min to enhance the solution penetration into tissues. Afterward, the assay tubes were incubated in a 33°C water bath for 30 min wrapped in aluminum to prevent light penetration. The reaction was stopped by adding 1 mL of 1% sulfanilamide in 2 mol L^–1^ HCl solution plus 1 mL aliquot of 0.05% naphthalenediamine solution. The results obtained were based on the nitrite (NO_2_^–^) standard curve and read in absorbance in a spectrophotometer at a wavelength of 540 nm. The NR activity in the fresh leaves was expressed as nM NO_2_^–^ h^–1^ g FW^–1^.

#### Obtaining the Crude Extract From Leaf Samples for Antioxidant Metabolism

To obtain the crude extract (Giannopolitis and Ries, 1977), sugarcane leaves (0.3 g) were ground with a mortar and pestle under liquid nitrogen and suspended in 5.0 mL of 0.1 M potassium phosphate buffer, pH 6.8, supplemented with 200 mg of polyvinylpolypyrrolidone (PVPP). Then, the samples were centrifuged for 15 min at 5,000 × *g* for 20 min. A 2-mL aliquot of supernatant was collected and stored in a freezer at (−80°C). From this extract, analyses of the total soluble protein and activity of the superoxide dismutase (SOD), catalase (CAT), and peroxidase (POX) were performed.

For proline determination (Bates et al., 1973), a second extract was obtained. Five hundred milligrams of the plant material (ground) was resuspended in 10.0 mL of sulfosalicylic acid (3% in deionized water). Then, the samples were centrifuged for 12 min at 4,000 × *g* at 4°C, and the supernatant was collected and stored in a freezer at −80°C for later determination of the proline content.

#### Total Soluble Protein Content

Total soluble protein determination occurred according to the methodology proposed by Bradford (1976). A 50-μL aliquot of the crude extract was mixed with 4,950 μL of Bradford’s solution and analyzed in a spectrophotometer at a wavelength of 595 nm. Bovine serum albumin (BSA) was used as a standard curve. The results and protein extract were used to determine the activities of SOD, CAT, POX, and proline.

#### Superoxide Dismutase Activity (EC 1.15.1.1)

The activity of SOD was determined according to the method proposed by Giannopolitis and Ries (1977). The reaction chamber under a 15-W fluorescent lamp at 25°C was used. A 50-μL aliquot of crude protein was mixed to 2 mL of potassium phosphate buffer solution at 50 mM and pH 7.8, plus 13 mM methionine, 75 μM NBT, 100 nM EDTA, and 2 μM riboflavin in 3.0 mL. The samples were vortexed, stored in a closed chamber free of any external light, and illuminated for 20 min in the reaction chamber. After that, the samples were homogenized and read in absorbance at a spectrophotometer wavelength of 560 nm, and the results are expressed as U SOD mg^–1^ protein.

#### Peroxidase Activity (EC 1.11.1.7)

The peroxidase (POD) activity was assayed according to the methodology of Peixoto et al. (1999). A 50-μL aliquot of crude extract was added in 4.95 mL of 25 mM potassium phosphate buffer, pH 6.8, containing 20 mM pyrogallol and 20 mM hydrogen peroxide (H_2_O_2_). After 1 min of incubation, the reaction was stopped with 0.5 mL of H_2_SO_4_. The absorbance reading was performed at 420 nm. A molar extinction coefficient of 2.47 mM^–1^ cm^–1^ was used for the calculation of the POD-specific activity and expressed in μK at μg protein^–1^.

#### Determination of Proline

The determination of the proline content occurred according to the method proposed by Bates et al. (1973). A 2.0-mL aliquot of the crude extract was mixed with 2.0 mL of acid ninhydrin and 2.0 mL of glacial acetic acid. Then, the mixture was heated at approximately 100°C for 60 min. After the cooling, the absorbance readings were performed at 520 nm. The results were applied to the standard curve of pure proline, and the values were expressed in μmol g fresh weight^–1^ (FW).

#### Sugar Concentration

Sugar fractions (reducing sugar, sucrose, and total soluble sugar concentration) on sugarcane leaves were extracted according to the modified method of Xu et al. (2015). The sugarcane leaf ground sample (0.1 g) was extracted with 80% (v/v) ethanol. The samples were mechanically stirred at 65°C for 1 h, followed by centrifugation at 10,000 × *g* for 15 min. The sugar fraction was determined spectrophotometrically according to the methods of Somogyi-Nelson (Nelson, 1944; Somogyi, 1952). The readings were performed by a spectrophotometer at 535-, 480-, and 620-nm wavelengths, respectively, for each sugar fraction. The ethanol-insoluble residue was used to determine the starch contents following the methodology proposed by Kuai et al. (2014) After evaporation of ethanol, 2 mL of deionized water was added into the samples and incubated at 100°C for 15 min. Starch hydrolyzation occurred by addition of 9.2 M and, later, 4.6 M HClO_4_ to the samples. Starch concentration was determined spectrophotometrically with anthrone reagent at 620-nm wavelength.

### Root Parameters

Prior to harvest, the root sampling occurred using a probe with 0.48 cm in diameter by 0.20 m in depth (same depth of the pots). The subsamples were washed under running water and scanned by a digitizer coupled to a computer with the WinRhizo software version 3.8-b (Regent Instruments Inc., QC, Canada), as described by Tennant (1975), to determine the root length. The values obtained were converted to m dm^–3^ from the pot volume and the probe. Afterward, samples were dried in a forced air oven at 60°C until reaching constant weight to determine the root dry weight. The values obtained were converted to g dm^–3^.

### Sugarcane Morphological Attributes

The sugarcane biometric and production parameters were assessed at harvest. The height of plants was determined by measuring with a graduated rule belonging to the soil up to the leaf +1. The stalk diameter was measured with a digital pachymeter, measuring the internode of the first third of the plant from the soil. The number of tillers occurred by the tillers count, discounting the mother plant. The leaf width was measured in the middle third of the leaf +1 with a graduated rule. Stalk fresh weight was determined after the harvest (stalks without leaves), and the stalk dry weight was determined after the samples were subjected to drying in a forced air oven at 65°C until the material was completely dried.

### Statistical Analysis

The normality and homoscedasticity of the data in all datasets were checked using the Anderson–Darling test, and homoscedasticity was analyzed with Levene’s test. Subsequently, the means were subjected to analysis of individual variance (ANOVA) by the *F*-test (*p* ≤ 0.05) and, when significant, were analyzed using Fisher’s protected least significant difference (LSD) at *p* ≤ 0.05.

## Results

### Crop Nutrition

Changes in sugarcane crop nutrition occurred (*p* < 0.01) only for N and S concentrations (Supplementary Table 2 and Supplementary Figure 2). The nitrogen leaf concentration from sugarcane plants inoculated with *T. asperellum* increased by 14% when established under optimal water availability, and 22% under drought stress (Supplementary Figure 2A). However, under drought stress, N uptake by non-inoculated plants reduced by 23%, and 18% in inoculated plants. Sulfur leaf concentration reduced in drought-stressed sugarcane plants, but *T. asperellum* inoculation increased the S acquisition by the plants, not differing from treatments without drought stress (Supplementary Figure 2F). The other macronutrients (P, K, Ca, and Mg) were not affected by the treatments (Supplementary Figures 2B–E).

### Photosynthetic Pigments and Gas Exchange

**FIGURE 2:**
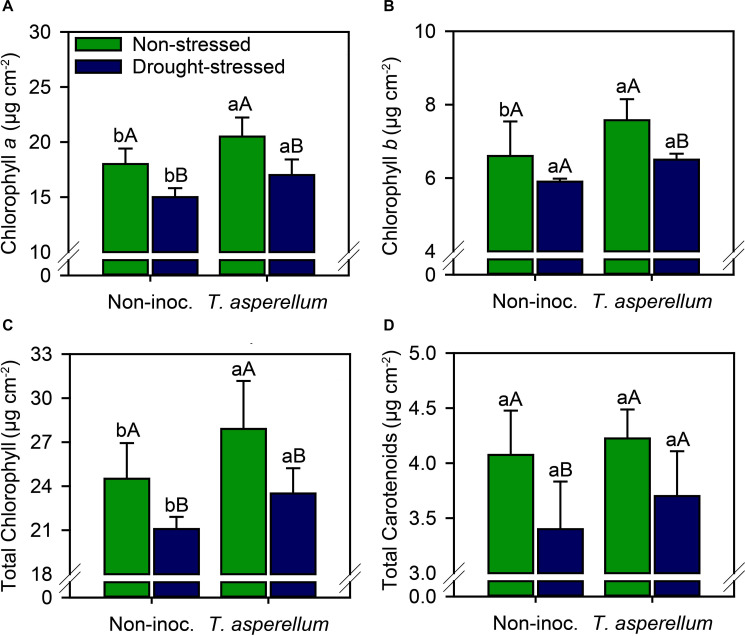
Chlorophyll *a* **(A)**, chlorophyll *b* **(B)**, total chlorophyll **(C)**, and total carotenoid **(D)** concentrations in sugarcane leaves according to the treatments. Different lowercase letters indicate significant differences between presence and absence of moderate drought stress, and different capital letters indicate significant differences between presence and absence of inoculation with *T. asperellum* by Fisher’s protected LSD test at *p* ≤ 0.05. Error bars express the standard error of the mean (*n* = 4).

Photosynthetic pigments changed according to the treatments (Supplementary Table 2 and Figure 2). In no-stressed plants, leaf chlorophyll concentration (Chl *a*, *b*, and total) increased (*p* < 0.01) in inoculated plants (14, 15, and 14%, respectively) (Figures 2A–C). In drought-stressed plants, *T. asperellum* inoculation also increased the chlorophyll concentration (13, 10, and 12%, respectively, for Chl *a*, *b*, and total), but the values found were lower than in non-stressed plants. *T. asperellum* inoculation did not change the carotenoid concentration, but under drought stress, non-inoculated plants presented 17% less carotenoids than plants established plants under adequate water availability; whereas in inoculated plants, drought stress reduced 12% the concentration of this pigment.

**FIGURE 3:**
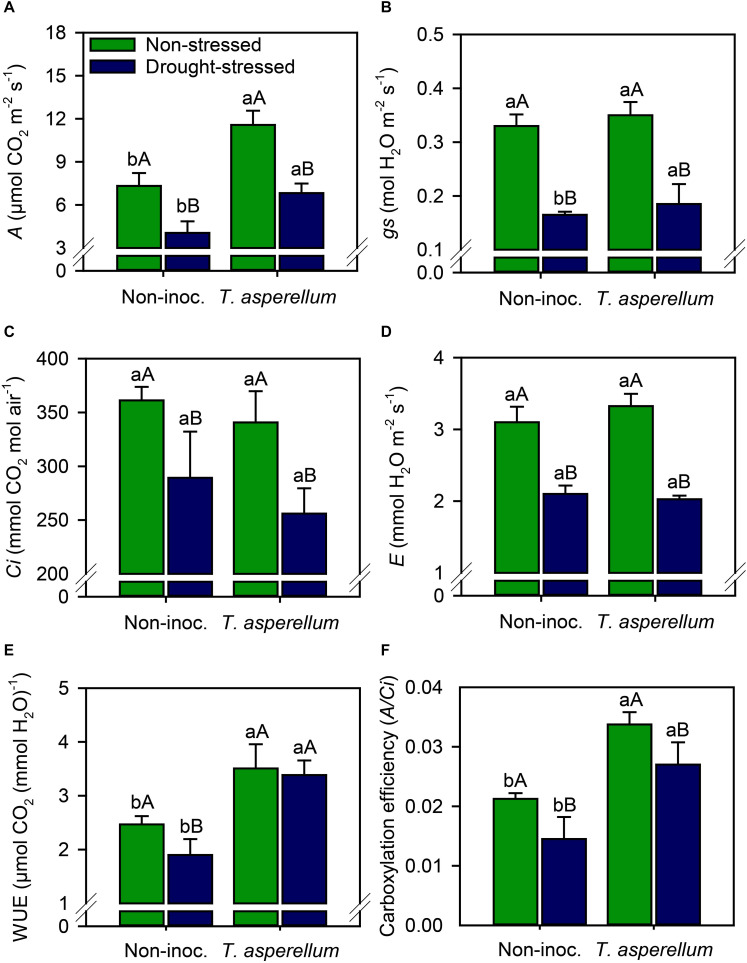
Net photosynthesis rate **(A)**, stomatal conductance **(B)**, substomatal CO_2_ concentration **(C)**, leaf transpiration **(D)**, water use efficiency **(E)**, and carboxylation efficiency **(F)** measured in sugarcane leaves according to the treatments. Different lowercase letters indicate significant differences between presence and absence of moderate drought stress, and different capital letters indicate significant differences between presence and absence of inoculation with *T. asperellum* by Fisher’s protected LSD test at *p* ≤ 0.05. Error bars express the standard error of the mean (*n* = 4).

*Trichoderma asperellum* inoculation increased (*p* < 0.01) the net photosynthetic rate (*A*) by 50% in non-stressed plants, and 68% in drought-stressed plants (Supplementary Table 2 and Figure 3A). Regarding the drought effects, *A* values reduced in 90% in non-stressed, and 70% in drought-stressed plants. Stomatal conductance (*gs*), internal CO_2_ concentration (*Ci*), and transpiration (*E*) did not change in non-stressed plants according to the inoculation treatment, but in drought-stressed plants, *T. asperellum* increased *gs* by 12% compared with non-inoculated plants (Figures 3B–D). In general, the drought stress effects in inoculated plants were lower than in non-inoculated one. Drought stress reduced by 50, 19, and 32% the *gs*, *Ci*, and *E*-values in non-inoculated plants and by 47, 24, and 39% in inoculated plants, respectively.

Considering the water use efficiency (WUE; *A*/*E* ratio), *T. asperellum* inoculation increased (*p* < 0.01) these values by 42% in non-stressed plants and by 78% in drought-stressed plants (Figure 3E). Drought stress did not change the WUE in inoculated plants, whereas it reduced by 23% this parameter in sugarcane plants that are non-inoculated. Regarding carboxylation efficiency (*A*/*Ci* ratio), drought stress reduced by 37 and 20% this parameter in non-inoculated and inoculated plants, respectively (Figure 3F). On the other hand, when sugarcane plants were inoculated, carboxylation efficiency increased by 59% in non-stressed plants and 86% in drought-stressed plants.

### Enzymatic Activity and Proline Concentration in Leaves

**FIGURE 4:**
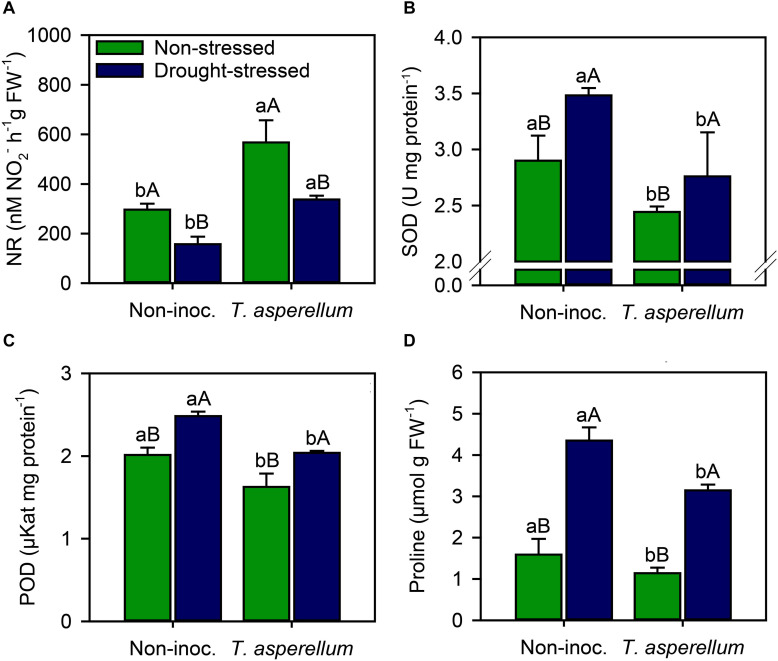
Activities of nitrate reductase **(A)**, superoxide dismutase **(B)**, peroxidase **(C)**, and proline concentrations **(D)** measured in sugarcane leaves according to the treatments. Different lowercase letters indicate significant differences between presence and absence of moderate drought stress, and different capital letters indicate significant differences between presence and absence of inoculation with *T. asperellum* by Fisher’s protected LSD test at *p* ≤ 0.05. Error bars express the standard error of the mean (*n* = 4).

Sugarcane plants inoculated with *T. asperellum* increased (*p* < 0.01) the NR activity in both non-stressed and drought-stressed plants by 91 and 114%, respectively, and drought stress most negatively affected the NR activity in non-inoculated plants (47%) than in inoculated one (40%) (Supplementary Table 2 and Figure 4A). Conversely, antioxidant enzymatic activity and proline concentration reduced (*p* < 0.01) when sugarcane plants were inoculated, in both water availability conditions. Drought stress in non-inoculated plants increased the activity of SOD (16%), POD (19%), and proline concentrations (63%), whereas in inoculated plants, the increase occurred by 11.5, 20, and 63% (Figures 4B–D). On the other hand, *T. asperellum* inoculation decreased the same parameters by 19% for SOD, 24% for POD, and 40% for proline in non-stressed plants, and by 26, 22, and 38%, respectively, in sugarcane plants under drought stress.

### Leaf Sugar Fractions

**FIGURE 5:**
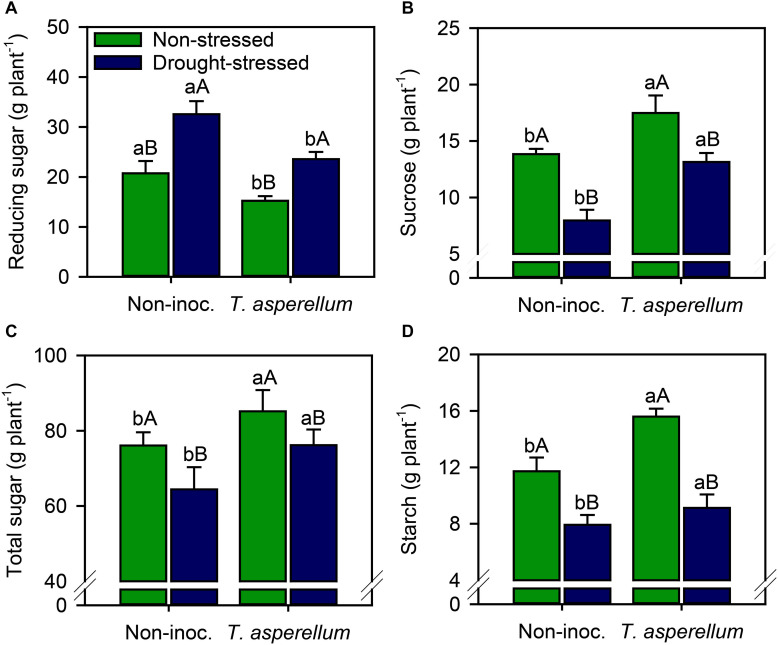
Reducing sugar **(A)**, sucrose **(B)**, total sugar **(C)**, and starch **(D)** concentrations in sugarcane leaves according to the treatments. Different lowercase letters indicate significant differences between presence and absence of moderate drought stress, and different capital letters indicate significant differences between presence and absence of inoculation with *T. asperellum* by Fisher’s protected LSD test at *p* ≤ 0.05. Error bars express the standard error of the mean (*n* = 4).

Reducing sugar concentration increased (*p* < 0.01) in drought-stressed plants, but sugarcane inoculated with *T. asperellum* reduced this value by 38% compared with non-inoculated plants (Supplementary Table 2 and Figure 5A). Increases (*p* < 0.01) occurred for sucrose (non-stressed = 26%; drought-stressed = 65%), total sugar (non-stressed = 12%; drought-stressed = 18%), and starch (non-stressed = 33%; drought-stressed = 15%) concentrations (Figures 5B–D). Notably, drought stress reduced sucrose (non-inoculated = 42%; inoculated = 24%), total sugar (non-inoculated = 15%; inoculated = 10%), and starch (non-inoculated = 32%; inoculated = 41%) concentrations compared with non-stressed plants; however, for sucrose and total sugar, drought stress effects were stronger in non-inoculated plants.

### Root and Morphological Attributes

**FIGURE 6:**
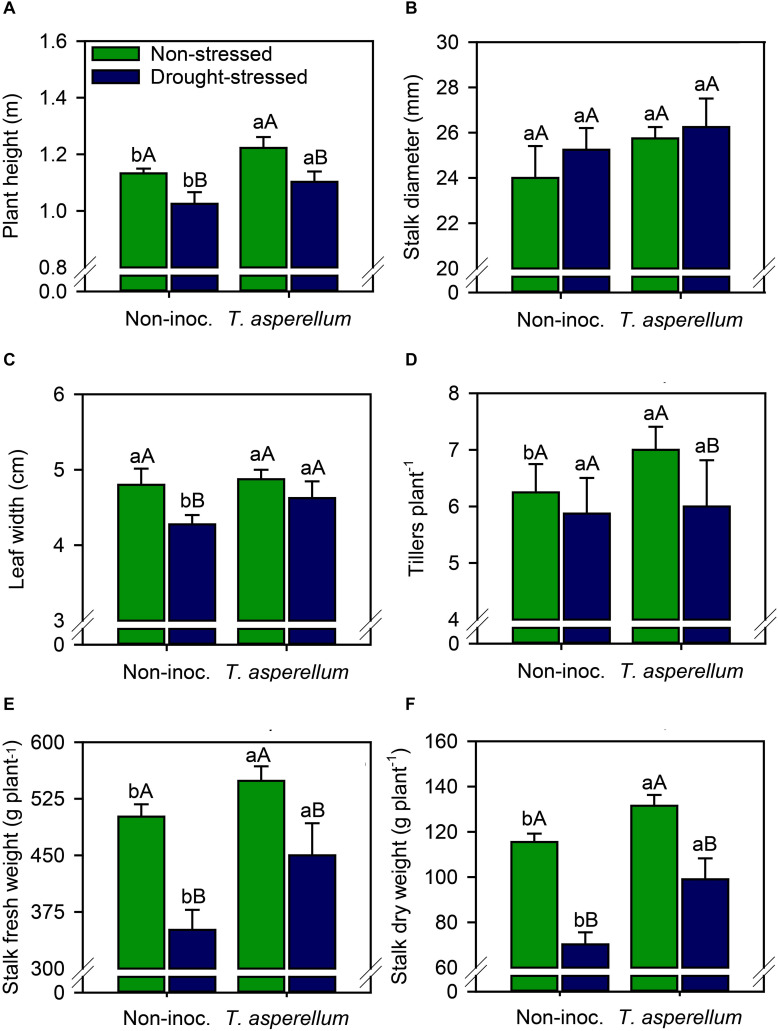
Plant height **(A)**, stalk diameter **(B)**, leaf width **(C)**, tillers plant^–1^ **(D)**, stalk fresh weight **(E)**, and stalk dry weight **(F)** of sugarcane plants according to the treatments. Different lowercase letters indicate significant differences between presence and absence of moderate drought stress, and different capital letters indicate significant differences between presence and absence of inoculation with *T. asperellum* by Fisher’s protected LSD test at *p* ≤ 0.05. Error bars express the standard error of the mean (*n* = 4).

All biometric and yield parameters were improved (*p* < 0.01) by the treatments, except stalk diameter (Supplementary Table 2 and Figure 6). *T. asperellum* inoculation in sugarcane plants increased plant height (non-stressed = 8.0%; drought-stressed = 7.6%) and in 8.2% the leaf width in drought-stressed plants (Figures 6A,C). Drought stress also reduced plant height (non-inoculated = 9.5%; inoculated = 9.8%) and by 11% the leaf width in non-inoculated plants. In addition, *T. asperellum* inoculation increased the number of tillers plant^–1^ (12%) in non-stressed plants (Figure 6D). As result of the combination of these parameters, stalk fresh and dry weight also improved (Figures 6E,F). Inoculated plants increased both fresh (non-stressed = 9.5%; drought-stressed = 28%) and dry (non-stressed = 14%; drought-stressed = 41%) weights of stalks. On the other hand, drought stress reduced stalk fresh weight (non-inoculated = 30%; inoculated = 18%) and stalk dry weight (non-inoculated = 39%; inoculated = 25%) compared with non-stressed plants.

**FIGURE 7:**
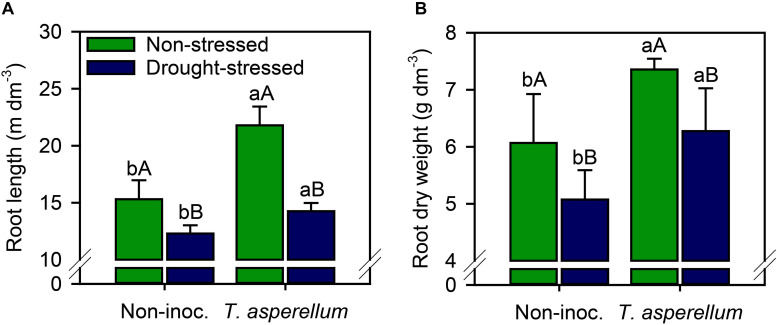
Root length **(A)** and root dry weight **(B)** of sugarcane plants according to the treatments. Different lowercase letters indicate significant differences between presence and absence of moderate drought stress, and different capital letters indicate significant differences between presence and absence of inoculation with *T. asperellum* by Fisher’s protected LSD test at *p* ≤ 0.05. Error bars express the standard error of the mean (*n* = 4).

Interestingly, *T. asperellum* inoculation not only improved the root length but also enhanced the root dry weight production (Supplementary Table 2 and Figure 7). In inoculated plants, increases (*p* < 0.01) occurred by root length (non-stressed = 42%; drought-stressed = 16%) and root dry weight (non-stressed = 21%; drought-stressed = 24%) (Figures 7A,B). Conversely, drought stress reduced the root length (non-inoculated = 20%; inoculated = 35%) and root dry weight (non-inoculated = 16%; inoculated = 15%).

## Discussion

Our understanding of *Trichoderma* spp. impacts on sugarcane nutrient uptake, plant physiology, and yield is limited, especially under environmental stress conditions. Previous studies have demonstrated that drought stress is one of the major environmental stress factors that cause biochemical alterations in plants, reduce plant growth, and decrease plant yield (Basu et al., 2016; Gupta et al., 2020). Therefore, our findings can help in understanding the effects of *T. asperellum* inoculation on sugarcane crop alleviating the drought stress effects in photosynthetic and antioxidant metabolism and their reflexes on stalk yield. Our results showed that *T. asperellum* inoculation improved not only sugarcane nutrition and agronomic parameters but also its physiological metabolism and yield (Figures 2–7). These findings are consistent with previous studies that reported that *Trichoderma* spp. can enhance yield and quality of tomato (Khoshmanzar et al., 2020), wheat (Zhang et al., 2016), rice (Doni et al., 2019; De Sousa et al., 2020), and maize (Fu et al., 2017; Estévez-Geffriaud et al., 2020) under abiotic stresses.

Generally, water deficiency reduces the nutrient uptake by the roots and transport from root to shoot (Ghassemi et al., 2018). However, the use of some microorganisms can assist in the acquisition of nutrients from the soil even under water restrictions (Khoshmanzar et al., 2020). Inoculation with *T. asperellum* presented higher N and S uptake in both non-stressed and drought-stressed plants (Supplementary Figure 2). The increase in N and S absorption can occur directly through the production of hydrolases (e.g., proteases and chitinases), which are responsible for degrading rhizospheric proteins and chitin and increasing the N and S uptake (Khoshmanzar et al., 2020), as well as indirectly, assisting in the supply of simple degradation compounds to heterotrophic nitrogen fixing microorganisms (Shoresh et al., 2010; Doni et al., 2019). In addition to the improvement in the nutrient acquisition from the soil solution, the N use efficiency seems to have been increased, given the greater activity of NR in inoculated plants (Figure 4A). Several evidences have shown that *Trichoderma* spp. are directly involved in N metabolism, mediated by the activation of NR in plants (Sherameti et al., 2005). The amount of nutrients applied to the plants was the same, so the increase in the nutrition of sugarcane plants, especially N, may be related to the increase in the efficiency of the use of this element. Nitrate reductase is the first enzyme in the nitrate assimilation pathway and probably is a determining factor in the efficiency of this process. Notwithstanding, *Trichoderma* spp. are widely recognized as phytohormones synthesizers (e.g., auxin) and hormone-like compounds called harzianolide and swollenin, substances that help along with auxin, in the expansion of the cell wall (Rajesh et al., 2016; De Sousa et al., 2020). These compounds enhance not only the shoot dry weight but also the root and rootlet development (De Sousa et al., 2020) involved in water and nutrient absorption, as reported in our study (Supplementary Figure 2). Therefore, sugarcane inoculated with *T. asperellum* performed better in terms of water and nutrient absorption by the roots compared to the non-inoculated plants, resulting in higher plant height and stalk production (Figure 6).

The benefits of *T. asperellum* inoculation in sugarcane plants are notorious, even under drought stress. The photosynthetic machinery of plants inoculated with this fungus has been improved, starting with the increase in chlorophyll concentrations (Figure 2). Chlorophyll concentration is widely used as an important indicator of abiotic tolerance in crops (Chutipaijit et al., 2011). As previously reported, *T. asperellum* changed the concentration of N, a nutrient directly involved in the composition of chlorophyll (Lawson and Flexas, 2020). In addition, plants exposed to drought stress usually show pronounced chlorosis due to rapid chlorophyll degradation (Zhang et al., 2016), leading to overall plant growth retardation. Our results showed that drought-induced stress significantly decreased chlorophyll concentration, but these values were reversed back to a level close to non-inoculated sugarcane plants grown under available water. In fact, we discovered that the chlorophyll concentration increased in sugarcane plants inoculated with *T. asperellum* whether or not subjected to drought stress. Our findings reinforce the important role of this microorganism in regaining the vigor of plants established in stressful environments.

As a result, *T. asperellum* also promoted benefits in photosynthesis rate (*A*), stomatal conductance (*gs*), water use efficiency (WUE), and carboxylation efficiency (Figure 3). Our results are in agreement with the findings of Bae et al. (2009) and De Sousa et al. (2020) who reported that plants inoculated with *Trichoderma* spp. reduced the drop on *A* and *gs* values, in addition to increasing the photosynthetic area of sugarcane leaves (Figure 6C). The increase in photosynthetic efficiency can also be seen in the increase in the levels of sugars and starch in sugarcane leaves (Figure 5). Although *E* and *Ci* values did not change by inoculation, sugarcane plants photosynthetically active and with stomatal conduction control showed higher WUE and carboxylation efficiency, since a higher rate of photosynthesis occurred for each unit of water that was evaporated by the stomata and CO_2_ that was carboxylated in the biochemical phase of photosynthesis. Stomatal conductivity can be altered by inoculation with *T. asperellum*, since this microorganism acts as an abscisic acid producer (Poveda, 2020). Abscisic acid is an important phytohormone related to the modulation of stomatal opening–closure movement (Zargar et al., 2017). These results are very relevant to sugarcane fields, as it is a semi-perennial crop, which is harvested in a cycle between 1 and 1.5 years and is subject to numerous periods of environmental stresses such as higher temperatures and water scarcity throughout its cycle (Marcos et al., 2018a). *T. asperellum* inoculation promoted an increase in the efficiency of gas exchange parameters; hence, the higher stalk and root weight in these plants was obtained (Figures 6, 7). These results revealed that the phytohormones and other similar compounds produced by this fungus are not the only mechanism to enhance root and stalk weight; that is, the relationship between water absorption and the losses from stomata and the increase in photosynthesis in inoculated than non-inoculated plants are also involved in enhancement in sugarcane below and aboveground development.

The reduction in chlorophyll degradation and the increase in the photosynthetic process may also be related to the increase in antioxidant systemic resistance in sugarcane plants inoculated with *T. asperellum* (Figure 4). The complex interaction between fungus and host may inhibit the production and accumulation of ROS by increasing the activity of enzymes and other antioxidant compounds in plant tissue (Brotman et al., 2013; Fu et al., 2017). The overproduction of ROS in plant tissues is considered a biochemical change responsible for damages to macromolecules and plant cellular structures under drought stress (Zargar et al., 2017); on the other hand, ROS production can also indicate signaling pathways (Foyer, 2018; Farooq et al., 2019). Under environmental stress conditions, for example, ROS production can function as a sensor that triggers repair mechanisms, assisting the plant in several processes that are linked to defense metabolism or cell growth (Foyer et al., 2017; Foyer, 2018). Although we did not evaluate ROS concentrations directly in our study, the antioxidant enzyme activity and, especially, the proline content indicated that in this case, the ROS produced in non-inoculated plus drought-stressed plants acted as causes of cell damage. To alleviate the ROS damages, plants produce the wide range of antioxidant defense mechanisms to detoxify free radical accumulation in their cells and protect them from oxidation (Zhang et al., 2016). The findings from our study demonstrated that drought stress induced plants to produce higher levels of SOD and POD activities, besides proline concentration, than the non-stressed plants, but *T. asperellum* reduced these values, probably due to the lower stress level in inoculated plants. Our results suggested that SOD and POD enzymes played a central protective role in the ROS scavenging in sugarcane plants treated with *T. asperellum*. In addition, drought stress increased proline biosynthesis, which enhances protein turnover (Khan et al., 2010). The proline compound is an important nitrogen source produced and used by plants to recover from abiotic stresses and restore their growth (Trotel et al., 1996). Karuppiah et al. (2019) studying cocultivation of *T. asperellum* and Bacillus amyloliquefaciens in wheat growth stated that this fungus induced the production of secondary metabolites, including proline, and increased the activity of antioxidant enzymes, both involved in increasing plant resistance against abiotic factors. In addition, another mechanism that may be involved in increasing resistance against drought stress is the participation of *Trichoderma* spp. in the regulation of resistance genes (Harman et al., 2004; De Sousa et al., 2020). In particular, *T. asperellum* is known as a modulator of defense gene expression during interaction with their host, regulating the pathway of jasmonic acid and salicylic acid (Segarra et al., 2007), both involved in increasing plant responses against environmental stresses (Segarra et al., 2007; Mastouri et al., 2010; De Sousa et al., 2020).

Interestingly, in drought-stressed plants, the reducing sugar increased, but *T. asperellum* inoculation decreased this parameter (Figure 5A). The opposite occurred for sucrose, total sugar, and starch concentrations (Figures 5B–D). This occurred because, under challenging environmental conditions, more complex carbohydrates (e.g., sucrose and starch) are generally remobilized to provide energy and carbon at times when photosynthesis may be potentially limited (Thalmann and Santelia, 2017), which results in an increase in reducing sugar concentrations (Figure 5). Carbohydrate portioning supports plant growth under stress, since carbohydrates act as osmoprotectants and signaling molecules, mitigating along with antioxidant metabolism the negative effect of the stress (Krasensky and Jonak, 2012; Thalmann and Santelia, 2017).

## Conclusion

In summary, sugarcane inoculated with *T. asperellum* altered the physiological parameters such as photosynthetic pigments, photosynthesis rate, stomatal conductance, water use efficiency and carboxylation efficiency, activation of NR enzyme, and antioxidant metabolism. This cascade effect culminated in sugarcane plant growth, tillering, and stalk yield even under drought stress. It is a significant result for sugarcane fields that constantly experience periods of water scarcity during the crop cycle. Inoculation is a feasible management tool, with promising and low-cost results that can increase the useful life of the sugarcane field and the farmer’s rentability. Our study evaluated the impact of inoculation on sugarcane seedlings; however, future studies should be carried out under field conditions to verify the viability and the need for the reinoculation of *T. asperellum* on sugarcane ratoons.

## Data Availability Statement

The original contributions presented in the study are included in the article/Supplementary Material, further inquiries can be directed to the corresponding author/s.

## Author Contributions

CC designed the experiment. DS and LM obtained and process the data. DS, JWB, and LM analyzed the data. DS, CC, and JWB wrote the manuscript with contribution of all co-authors. All authors contributed to the article and approved the submitted version.

## Conflict of Interest

The authors declare that the research was conducted in the absence of any commercial or financial relationships that could be construed as a potential conflict of interest.
