# Supplementary material for: Trichoderma asperellum Inoculation as a Tool for Attenuating Drought Stress in Sugarcane
Source: Front Plant Sci. 2021 Apr 15;12:645542. doi: 10.3389/fpls.2021.645542 (PMC8082249; doi:10.3389/fpls.2021.645542)
Supplement: Supplementary file 1 [file Data_Sheet_1.pdf]

# SUPPLEMENTARY MATERIAL

## *Trichoderma asperellum* inoculation as a tool for attenuating drought stress in sugarcane

Daniele Scudeletti[1†], Carlos Alexandre Costa Crusciol[1*†], João William Bossolani[1†], Luiz Gustavo Moretti[1], Letusa Momesso[1], Brenda Servaz Tubaña[2], Sérgio Gustavo Quassi de Castro[3], Elisa Fidêncio De Oliveira[1], Mariângela Hungria[4**]

[1] São Paulo State University (UNESP), College of Agricultural Sciences, Department of Crop Science, Botucatu, 18610-034, São Paulo, Brazil;
[2] School of Plant, Environmental, and Soil Sciences, LSU AgCenter, 104 Sturgis Hall, Baton Rouge, LA 70803, US;
[3] AgroQuatro-S applied agronomic experimentation, 6 Avenue 883, Orlândia, 14620-000, São Paulo, Brazil;
[4] Embrapa Soybean. Carlos João Strass Highway, Post Office Box: 231, Londrina, PR 86001-970, Brazil;

[†] These authors contributed equally to this work.

**Correspondence**
[*]carlos.crusciol@unesp.br (C.A.C. Crusciol)
[**]mariangela.hungria@embrapa.br (M. Hungria)

**Table S1.** Physical-chemical analysis of the soil before the beginning of the experiment.

| Sand | Clay | Silt | Soil texture | Soil density |
|---|---|---|---|---|
| | (g kg$^{-1}$) | | | (g cm$^{-3}$) |
| 810 | 141 | 49 | Sandy | 1.46 |

| pH | SOM | $P_{resin}$ | $K^+$ | $Ca^{2+}$ | $Mg^{2+}$ | SB | CTC | BS |
|---|---|---|---|---|---|---|---|---|
| ($CaCl_2$) | (g dm$^{-3}$) | (mg dm$^{-3}$) | | | (mmol$_c$ dm$^{-3}$) | | | |
| 4.1 | 5 | 2 | 0.31 | 4 | 1 | 6 | 22 | 25 |

| S | $Al^{3+}$ | H+Al | Fe | Cu | Mn | Zn | B |
|---|---|---|---|---|---|---|---|
| (g dm$^{-3}$) | (mmol$_c$ dm$^{-3}$) | | | | (mg dm$^{-3}$) | | |
| 9 | 12 | 17 | 88 | 1.1 | 9.2 | 1.2 | 0.7 |

**Table S2.** Statistical parameters of ANOVA test (p≤0.05) for all sugarcane variables studied.

| Treatments | ANOVA (*F probability)* | | | | | |
|---|---|---|---|---|---|---|
| | | | Crop nutrition | | | |
| | N | P | K | Ca | Mg | S |
| Inoculation (I) | 0.0213 | 0.1070 | 0.8642 | 0.4264 | 0.1833 | 0.0322 |
| Drought Stress (DS) | <0.001 | 0.5263 | 0.1062 | 0.6119 | 0.0760 | 0.1523 |
| I X DS | 0.0462 | 0.6330 | 0.7586 | 0.5247 | 0.3104 | 0.0425 |
| | | | Photosynthetic pigments | | | |
| | Chl *a* | Chl *b* | Total chlorophyll | Total carotenoids | | |
| Inoculation (I) | 0.0016 | <0.001 | <0.001 | 0.0698 | | |
| Drought Stress (DS) | 0.0022 | <0.001 | <0.001 | 0.0075 | | |
| I X DS | 0.0123 | 0.0495 | 0.0144 | 0.0500 | | |
| | | | Gas exchange | | | |
| | *A* | *gs* | *Ci* | *E* | WUE | *A*/*Ci* |
| Inoculation (I) | <0.001 | 0.0021 | 0.220 | 0.0783 | <0.001 | <0.001 |
| Drought Stress (DS) | <0.001 | <0.001 | <0.001 | <0.001 | 0.0032 | <0.001 |
| I X DS | 0.0037 | 0.0427 | 0.0509 | 0.0602 | 0.0352 | 0.0185 |
| | | | Sugars concentration | | | |
| | Reducing Sugar | Sucrose | Total sugar | Starch | | |
| Inoculation (I) | <0.001 | <0.001 | 0.0012 | <0.001 | | |
| Drought Stress (DS) | <0.001 | <0.001 | 0.0012 | <0.001 | | |
| I X DS | 0.0045 | 0.0074 | 0.0372 | <0.001 | | |
| | | | Enzymes activity | | | |
| | NR | SOD | POD | Proline | | |
| Inoculation (I) | <0.001 | <0.001 | <0.001 | <0.001 | | |
| Drought Stress (DS) | <0.001 | <0.001 | <0.001 | <0.001 | | |
| I X DS | 0.0487 | 0.0237 | 0.0492 | <0.001 | | |
| | | | Root development | | | |
| | Root length | Root dry weight | | | | |
| Inoculation (I) | <0.001 | 0.0020 | | | | |
| Drought Stress (DS) | <0.001 | 0.0066 | | | | |
| I X DS | 0.0010 | 0.0015 | | | | |
| | | | Biometric parameters | | | |
| | Plant height | Stalk diameter | Leaf width | Tillers plant$^{-1}$ | Stalk fresh weight | Stalk dry weight |
| Inoculation (I) | <0.001 | 0.0047 | 0.0320 | 0.0126 | <0.001 | <0.001 |
| Drought Stress (DS) | <0.001 | 0.0090 | 0.0041 | 0.0126 | <0.001 | <0.001 |
| I X DS | 0.0325 | 0.0412 | 0.0320 | 0.0404 | 0.0135 | 0.0076 |

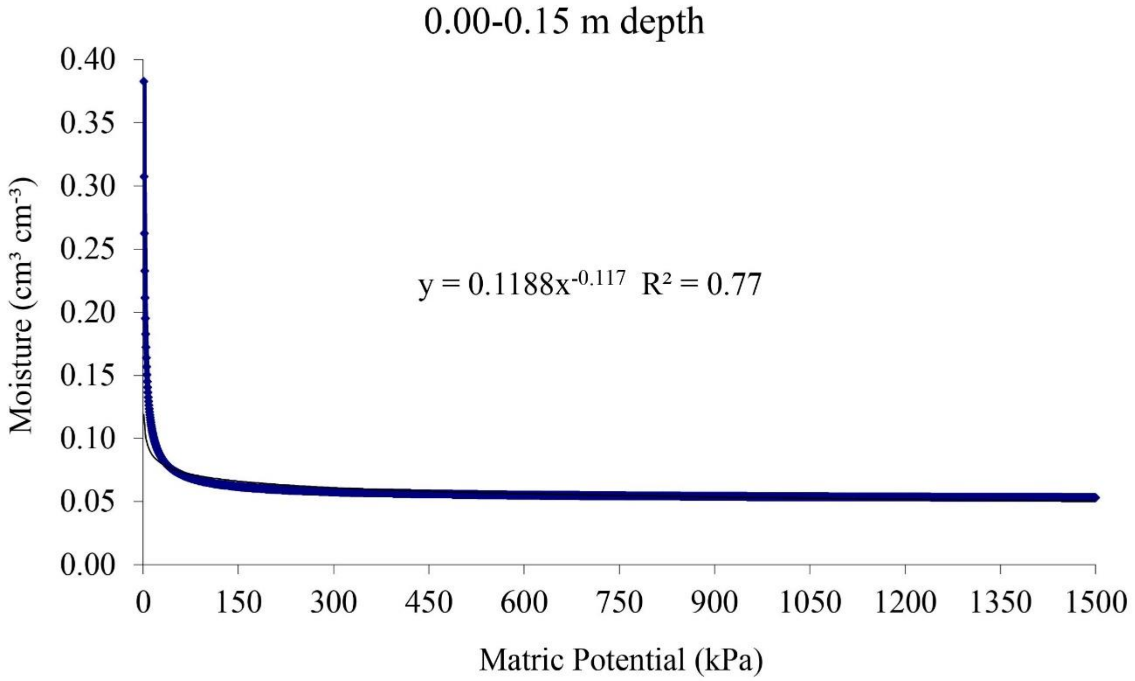


**Figure S1.** Soil water retention curve from soil used in the present study.

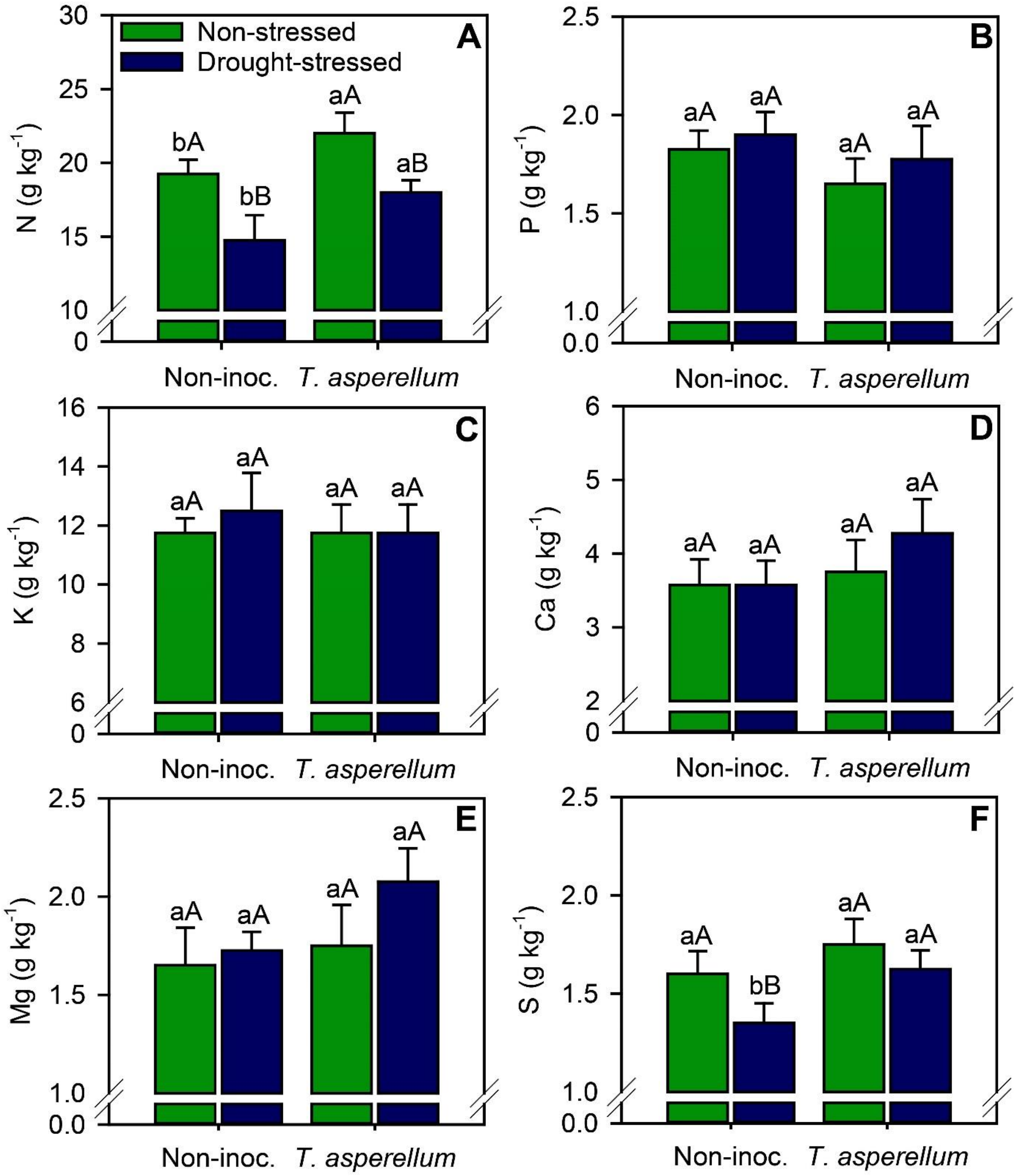


**Figure S2.** Concentration of nitrogen (a), phosphorus (b), potassium (c), magnesium (d), calcium (e), and sulfur (f) in sugarcane leaves according to the treatments. Different lower-case letters indicate significant differences between presence or absence of moderate drought stress, and different capital letters indicate significant differences between presence or absence of inoculation with *T. asperellum* by Fisher's protected LSD test at $p \leq 0.05$. Error bars express the standard error of the mean ($n = 4$).
